# Construction of TF-lncRNA-miRNA-mRNA Regulatory Network Affecting Sow Reproduction Based on QTLs for Corpus Luteum Number

**DOI:** 10.3390/ani16111693

**Published:** 2026-06-01

**Authors:** Miaomiao Wang, Min Lu, Yajie Gao, Chenxu Wang, Tengteng Xu, Chen Yang, Yong Liu

**Affiliations:** Anhui Province Key Laboratory of Embryo Development and Reproductive Regulation, Fuyang Normal University, Fuyang 236037, China; wmm@fynu.edu.cn (M.W.); 2024211313@stu.fynu.edu.cn (M.L.); 2025211319@stu.fynu.edu.cn (Y.G.); 2025221301@stu.fynu.edu.cn (C.W.); 202501012@fynu.edu.cn (T.X.); yangchen09@yeah.net (C.Y.)

**Keywords:** corpus luteum number, QTL, TF-lncRNA-miRNA-mRNA network, sow reproduction

## Abstract

The corpus luteum number in sows is a key reproductive indicator for measuring ovulation rate and reproductive efficiency. Its formation is precisely regulated by a complex gene regulatory network composed of multi-level molecular interactions. To systematically elucidate the molecular basis of this trait, this study comprehensively analyzed genes located within QTL for corpus luteum number. This approach identified a series of key regulatory molecules specifically expressed in the ovary, including transcription factors (TFs), long non-coding RNAs (lncRNAs), and microRNAs (miRNAs). Using bioinformatics methods to predict the target genes of candidate miRNAs, combined with functional enrichment analysis, revealed that these target genes were significantly enriched in multiple core reproductive pathways closely related to cell proliferation, differentiation, and hormone regulation, including the ErbB signaling pathway, PI3K-Akt signaling pathway, and TGF-beta signaling pathway. Based on the above findings, this study ultimately constructed a TF-lncRNA-miRNA-mRNA network, which is associated with QTL for corpus luteum number. Furthermore, key genes were validated via quantitative real-time PCR (qRT-PCR). Significant positive correlations were identified between the transcription factor *NEUROG2* and lncRNA *LOC102167554*, along with its potential target gene *ESRP1*, as well as between transcription factor *SNAI2* and lncRNAs (*LOC102167554*, *LOC102167796*) and their potential target genes (*FXR*, *ERBB4*). In addition, the functional validation results showed that the interference of *LOC102167554* significantly reduced the proliferation ability of sGCs. These key genes represent potential targets for genetic improvement of sow reproduction.

## Simple Summary

This study systematically constructed a potential multi-level regulatory network involving TF-lncRNA-miRNA-mRNA interactions centered on candidate genes within quantitative trait loci (QTL) for corpus luteum number in sows. Three potential reproductive regulatory axes were identified: *NEUROG2*/*LOC102167554*/*ESRP1*, *SNAI2*/*LOC102167554*/*FXR*, and *SNAI2*/*LOC102167796*/*ERBB4*. This provides new insights for elucidating the molecular mechanisms underlying follicular development in sows. In addition, a functional lncRNA, *LOC102167554*, that regulates the proliferation of sow granulosa cells (sGCs) was also identified.

## 1. Introduction

Reproductive traits in sows are critical economic characteristics in swine production, directly determining the overall productivity and economic efficiency of the industry. Sow fertility is a complex quantitative trait influenced by multiple factors, including ovulation rate, nutritional supply, and rearing environment [1,2,3]. Among these, ovulation rate is the core factor determining the upper limit of litter size. Following ovulation in sows, follicular structures transform into a corpus luteum. Consequently, the corpus luteum number serves as a critical reproductive indicator for evaluating ovulation rate and reproductive performance. In recent years, with the widespread application of technologies such as genome-wide association studies and high-throughput sequencing in livestock, multiple QTLs for corpus luteum number have been identified in sows. Furthermore, studies have revealed that candidate genes within reproductive QTLs regulate reproductive traits and cellular functions (such as granulosa cell proliferation, apoptosis, and estradiol (E2) synthesis) [1,4], thereby influencing sow reproductive performance. Therefore, screening and validating candidate genes within QTLs associated with sow corpus luteum number is crucial for elucidating the regulatory mechanisms of ovulation, which will ultimately facilitate the improvement of sow reproductive performance through molecular breeding.

The advent of high-throughput sequencing technologies and the development of systems biology have ushered research on the genetic regulation of reproductive traits into an era of multi-omics integration. It is now recognized that the manifestation of complex traits is often governed by intricate regulatory networks comprising multi-level genes (including TFs, lncRNAs, miRNAs, and mRNAs). Among these, lncRNAs, a class of RNA molecules exceeding 200 nt in length with limited protein-coding potential, have emerged as a hotspot in life science research in recent years, due to their unique structural characteristics and regulatory potential. Accumulating evidence indicates that lncRNAs are extensively involved in diverse critical biological processes, including cell proliferation, apoptosis, and both transcriptional and post-transcriptional regulation [5,6,7,8]. Additionally, numerous lncRNAs exhibit differential expression in healthy atretic follicles and in the ovaries of high- and low-fertility sows [9,10]. Importantly, several of these lncRNAs have been functionally demonstrated to regulate reproductive processes in swine [1,11]. These findings highlight lncRNAs as promising candidate targets for modulating sow reproductive performance.

miRNAs are a class of non-coding RNAs, typically 21–24 nt in length, that commonly suppress gene expression by binding to the 3′ untranslated region (3′UTR) of target mRNAs through base pairing [12]. LncRNAs and miRNAs are functionally interconnected. In the cytoplasm, lncRNAs can serve as molecular sponges that sequester miRNAs, thereby regulating the expression of miRNA target mRNAs through a competing endogenous RNA (ceRNA) mechanism. For instance, lncRNA *lnc2300* acts as a molecular sponge for miR-365-3p, thereby derepressing its target *CYP11A1* and inhibiting the apoptosis of porcine granulosa cells [13]. Similarly, lncRNA *NORFA* competitively binds miR-126, preventing the degradation of its target gene *TGFBR2* and inhibiting the apoptosis of porcine granulosa cells [11].

As the primary executors of biological functions, coding genes not only directly contribute to physiological processes as structural or functional proteins but also generate diverse protein isoforms with distinct roles through post-transcriptional regulatory mechanisms, such as alternative splicing [14,15]. This molecular plasticity enables precise adaptation of gene expression to the specific demands of various reproductive stages. Additionally, coding genes can encode TFs that activate or inhibit the transcription of downstream genes (including mRNA, lncRNA, and miRNA) by binding to specific cis-elements, thereby participating in reproduction-related processes [16]. Notably, some of the protein isoforms derived from coding genes may serve as TFs, which further expands the regulatory complexity of gene expression in reproductive processes [17]. Although these regulatory factors have been identified as involved in regulating the reproductive processes of sows, their systematic interaction networks within QTL regions associated with reproductive traits remain poorly understood. Therefore, it is necessary to further investigate the mutual regulatory relationships among lncRNAs, miRNAs, mRNAs, and TFs in the reproductive regulation of sows. The current study aims to construct a TF–lncRNA–miRNA–mRNA regulatory network for sow reproductive regulation, focusing on the key QTL associated with corpus luteum number and reproductive traits.

This study systematically screened key TFs, lncRNAs, and miRNAs specifically expressed in the ovary by integrating QTLs for corpus luteum number with multi-tissue transcriptomic data. Bioinformatics methods were employed to predict target genes of candidate miRNAs. Kyoto Encyclopedia of Genes and Genomes (KEGG) and Gene Ontology (GO) analysis revealed significant enrichment of these target genes in signaling pathways closely associated with reproductive regulation, such as the ErbB signaling pathway, TGF-beta signaling pathway, and others. The PI3K-Akt signaling pathway and the TGF-beta signaling pathway are pathways critically involved in reproductive regulation. Based on these findings, a comprehensive TF-lncRNA-miRNA-mRNA regulatory network was constructed, elucidating the multi-level molecular interactions involved in sow reproduction. To further assess the reliability of this predicted network, expression correlations among key nodes were validated using qRT-PCR. This study provides a novel theoretical framework for deepening the understanding of the molecular regulation of sow reproduction.

## 2. Materials and Methods

### 2.1. Bioinformatic Analysis

QTL data were obtained from the pigQTLdb (https://www.animalgenome.org/cgi-bin/QTLdb/SS/index (accessed on 1 March 2021)). Subsequently, QTLs closely associated with sow reproductive traits were screened and filtered. Genes fully located within these candidate QTL regions were finally defined as potential candidate genes. LncRNA tissue expression data were downloaded from NCBI (https://www.ncbi.nlm.nih.gov/). Subcellular localization of lncRNAs was predicted online using lncLocator (http://www.csbio.sjtu.edu.cn/bioinf/lncLocator/ (accessed on 8 December 2025)). The binding interaction between lncRNA and miRNA was predicted via the RNAhybrid (http://bibiserv.techfak.uni-bielefeld.de/rnahybrid/ (accessed on 4 January 2026)). The interaction network was created using Cytoscape_v3.10.1. Potential miRNA target genes were predicted via Targetscan 8.0 (https://www.targetscan.org/vert_80/ (accessed on 2 January 2026)) and miRDB (https://mirdb.org/). Draw Venn Diagram (http://bioinformatics.psb.ugent.be/webtools/Venn/ (accessed on 4 January 2026)) was performed to screen the common target genes jointly predicted by the TargetScan and miRDB (Target Score > 80) databases. The potential functions of candidate miRNAs and their associated signaling pathways were analyzed by DAVID (https://davidbioinformatics.nih.gov/ (accessed on 4 January 2026)) through GO and KEGG analyses. Genes located entirely within the QTLs were identified as potential candidate genes.

### 2.2. Animals, Samples, and Ethics

Ovaries of Duroc × Landrace × Yorkshire sows sexually mature were obtained from a local slaughterhouse. These sows (180 days old) were in good condition, sexually mature, and not in estrus. Experiments were approved by the Animal Ethics Committee of Fuyang Normal University (Fuyang, China).

### 2.3. Follicle Isolation

Fresh sow ovaries were washed in 37 °C saline solution after collection. Following washing, they were placed in an insulated container containing 37 °C saline solution and transported back to the laboratory within 1 h. Follicles were separated using established methods [4]. Specifically, fresh ovaries obtained from the slaughterhouse are washed several times with 37 °C saline solution and then placed in a clean petri dish (containing 37 °C saline solution). The ovarian tunica albuginea was gently incised with a scalpel to expose follicles measuring 3–5 mm in diameter. The superficial layer of the tunica albuginea was then removed using surgical forceps. Subsequently, the lower layer of the tunica albuginea surrounding the ovarian tunica was removed with surgical forceps, and follicles were gently peeled away. After follicular fluid removal, follicles were subjected to RNA isolation and reverse transcription, followed by qRT-PCR to validate the expression correlation of candidate genes.

### 2.4. RNA Isolation and qRT-PCR

Total RNA extraction and reverse transcription were conducted using TRIzol reagent (Vazyme, Nanjing, China) and SweScript All-in-One Blue RT SuperMix for qPCR (Servicebio, Wuhan, China), respectively. For RNA samples with an OD260/280 ratio greater than 1.8, we used 1000 ng RNA for reverse transcription. RNA quantification was conducted using 2× Universal Blue SYBR Green qPCR Master Mix (Servicebio, Wuhan, China). qRT-PCR reaction conditions are as follows: 1. pre-denaturation: 95 °C for 30 s; 2. cyclic reaction (40 cycles): 95 °C for 15 s and 60 °C for 30 s; and 3. melting curve: 60 °C for 1 min and 95 °C for 1 s. The 2^−ΔΔCT^ method was used to estimate the relative transcript levels. GAPDH was the internal control. Primers are listed in Table S1.

### 2.5. Plasmids and Mimics

Reporter plasmids of the *LOC102167554* and *ERBP1* transcripts containing the response elements of miR-199a-3p were obtained from Tsingke (Shanghai, China). Mimics for miRNAs were ordered from Generay (Shanghai, China).

### 2.6. Cell Preparation and Transfection

The sGCs isolated from 3–5 mm ovarian follicles were cultured in plates with DMEM/F12 medium (Gibco, Carlsbad, CA, USA) supplemented with 15% fetal bovine serum (FBS) (Ozfan, Nanjing, China) and 1% penicillin-streptomycin (PS) (Gibco, Carlsbad, CA, USA). HEK-293T cells were maintained in high-glucose DMEM (Gibco, Carlsbad, CA, USA) containing 10% FBS and 1% PS. All cells were incubated at 37 °C with 5% CO_2_. The miRNA mimics were transfected into cells using Lipofectamine 3000 (Invitrogen, Carlsbad, CA, USA).

### 2.7. Cell Proliferation Assay

Cell proliferation of sGCs was evaluated using the CCK-8 Cell Counting Kit (Vazyme, Nanjing, China). Briefly, sGCs were seeded in 96-well plates at a density of 1 × 104 cells/well with 90 μL of culture medium, and six biological replicates were set for each group. At 0, 24, 48, and 72 h post-transfection, 10 μL of CCK-8 reagent was added to each well, followed by incubation at 37 °C for 1 h. Subsequently, the absorbance of each sample was detected at 450 nm using a Multiskan Go spectrophotometer (Thermo Fisher, Waltham, MA, USA).

### 2.8. Statistics

We used GraphPad v8.0 (San Diego, CA, USA) for statistical analysis. The correlation coefficient was calculated using Pearson’s test. Significance was assessed using Student’s *t*-test with a two-tailed distribution. *p*-values of <0.05 were considered statistically significant.

## 3. Results

### 3.1. Identification of Potentially Functional lncRNAs in QTLs for Corpus Luteum Number

**Figure 1:**
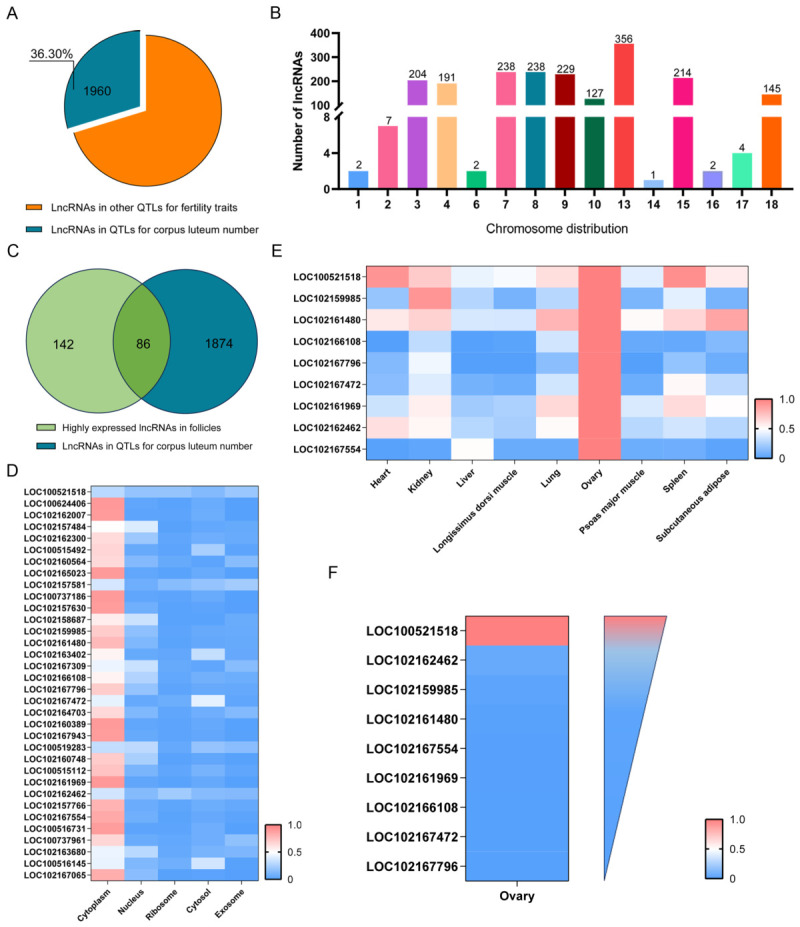
Identification of potentially functional lncRNAs in QTLs for corpus luteum number. (**A**) The proportion of lncRNAs located in QTLs for corpus luteum number relative to reproductive lncRNAs. (**B**) Chromosome distribution of the lncRNAs in QTLs for corpus luteum number. (**C**) Diagram depicting lncRNAs located in QTLs for corpus luteum number and highly expressed in follicles. (**D**) Prediction of subcellular localization of candidate lncRNAs using the online tool lncLocator (http://www.csbio.sjtu.edu.cn/bioinf/lncLocator/ (accessed on 8 December 2025)). (**E**,**F**) Tissue expression profile of ovarian highly expressed lncRNAs. LncRNA tissue expression data are sourced from the NCBI database (https://www.ncbi.nlm.nih.gov/). The data has been normalized.

The ovulation rate, for which corpus luteum number is a key indicator, is a critical economic trait in sow reproduction. Previous studies have established that lncRNAs located within QTLs for corpus luteum number can influence reproductive traits by regulating key physiological processes [1]. We conducted a systematic analysis of lncRNAs situated within QTLs for fertility traits. The results revealed that 36.3% of these lncRNAs reside within QTLs for corpus luteum number (Figure 1A), suggesting these lncRNAs may regulate sow ovulation. Analysis of the chromosomal distribution revealed that the candidate lncRNAs, while spanning 15 autosomes, exhibited a non-uniform pattern (Figure 1B). A pronounced enrichment was observed on chromosome 13, whose QTL region contained the highest number of these lncRNAs (356), suggesting this locus as a potential hotspot for genetic elements influencing sow reproductive performance. Follicles serve as the fundamental functional units for oocyte development and maturation, and their developmental status directly determines key reproductive traits, including ovulation rate. Given their physiological importance, we focused our subsequent analysis on lncRNAs highly expressed in follicular tissue, building on our previous findings [1]. This screening approach identified 84 lncRNAs that were both highly expressed in follicles and located within QTLs for luteum number (Figure 1C).

Subcellular localization is a key determinant of lncRNA function and mechanisms [18]. We predicted the subcellular localization of 84 candidate lncRNAs. The results showed that 34 lncRNAs were primarily expressed in the cytoplasm (Figure 1D, Table S2). To further refine the pool of functionally relevant lncRNAs, we analyzed the tissue expression profiles of the 34 cytoplasmic candidates. This analysis identified nine lncRNAs with predominant expression in ovarian tissue (Figure 1E,F). Importantly, none of these nine lncRNAs have been previously associated with sow reproductive traits, representing a novel set of candidate molecules with significant potential for future functional studies.

### 3.2. Identification of Potentially Functional miRNAs in QTLs for Corpus Luteum Number

**Figure 2:**
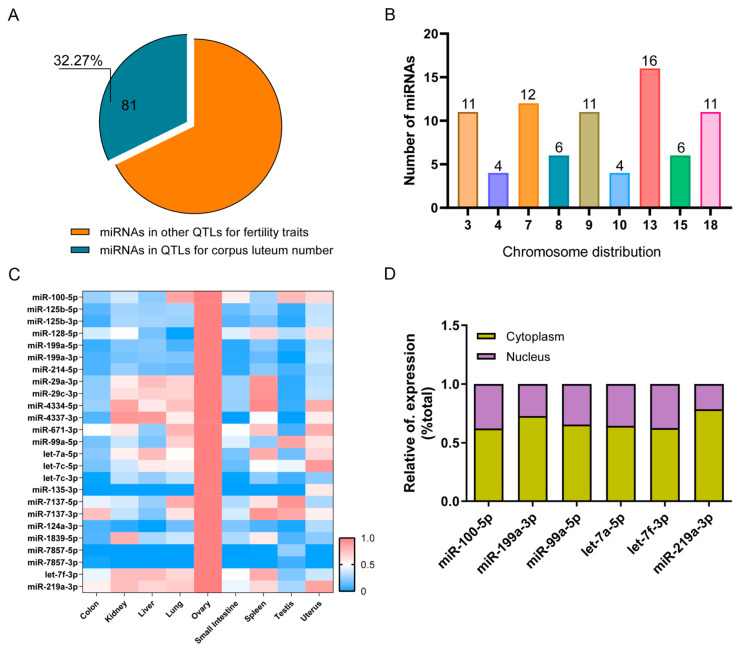
Identification of potentially functional miRNAs in QTLs for corpus luteum number. (**A**) The proportion of miRNAs located in QTLs for corpus luteum number relative to reproductive lncRNAs. (**B**) Chromosome distribution of the miRNAs in QTLs for corpus luteum number. (**C**) The heatmap displays miRNAs located in QTLs for corpus luteum number and highly expressed in ovaries. (**D**) Levels of miRNAs in nuclear and cytoplasmic fractions isolated from sGCs. Data for miRNA subcellular localization were obtained from a previous report [19].

To investigate the potential involvement of lncRNA-miRNA interactions in the regulation of ovulation, we conducted a statistical analysis of miRNAs localized within the QTL for corpus luteum number. The results indicate that 32.27% of reproduction-related miRNAs are located within QTLs for corpus luteum number (Figure 2A, Table S3), with these candidate miRNAs distributed across nine autosomes (Figure 2B). Further tissue expression profiling revealed that 25 of these miRNAs are primarily expressed in ovarian tissue, suggesting their potential involvement in regulating reproductive processes, such as ovulation (Figure 2C). Given that cytoplasmic lncRNAs regulate miRNAs through the ceRNA mechanism, which represents the most classical and prevalent interaction pattern, we performed subcellular localization analysis on the aforementioned miRNAs. This revealed that six miRNAs are primarily expressed in the cytoplasm (Figure 2D). Consequently, these were selected as key candidate molecules for subsequent studies to thoroughly elucidate their potential ceRNA regulatory networks.

### 3.3. The Construction of the lncRNA-miRNA Network

**Figure 3:**
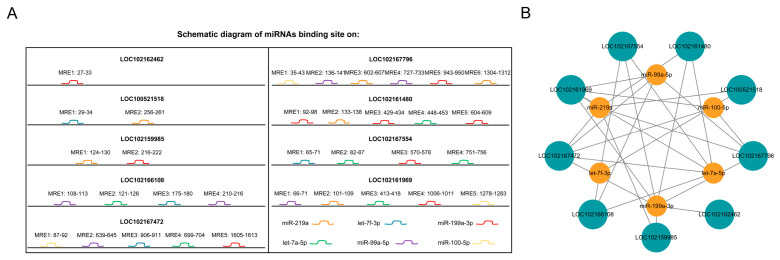
The construction of the lncRNA-miRNA network. (**A**) Schematic diagram of candidate lncRNA-miRNA interactions. (**B**) lncRNA-miRNA interaction network. Blue and yellow circles represent lncRNAs and miRNAs.

To determine whether the nine candidate lncRNAs could serve as ceRNAs for the six candidate miRNAs, their potential binding capacity was predicted using the online platform RNAhybrid. *LOC102162462*, *LOC100521518*, *LOC102159985*, *LOC102166108*, *LOC102167554*, *LOC102167472*, *LOC102161480*, *LOC102161969*, and *LOC102167796* were identified as containing 1, 2, 2, 4, 4, 5, 5, 5, and 6 putative miRNA response elements (MREs), respectively (Figure 3A). To further visualize the regulatory relationships between lncRNAs and miRNAs, we constructed a lncRNA-miRNA interaction network using Cytoscape (Figure 3B). This network reveals that certain lncRNA nodes are connected to multiple miRNAs, while several miRNAs are also linked to multiple lncRNAs. This indicates that complex regulatory interactions may exist among multiple lncRNAs and miRNAs, suggesting the presence of potential negative feedback regulatory mechanisms between them.

### 3.4. Prediction and Function Analysis of the miRNA Target Genes

**Figure 4:**
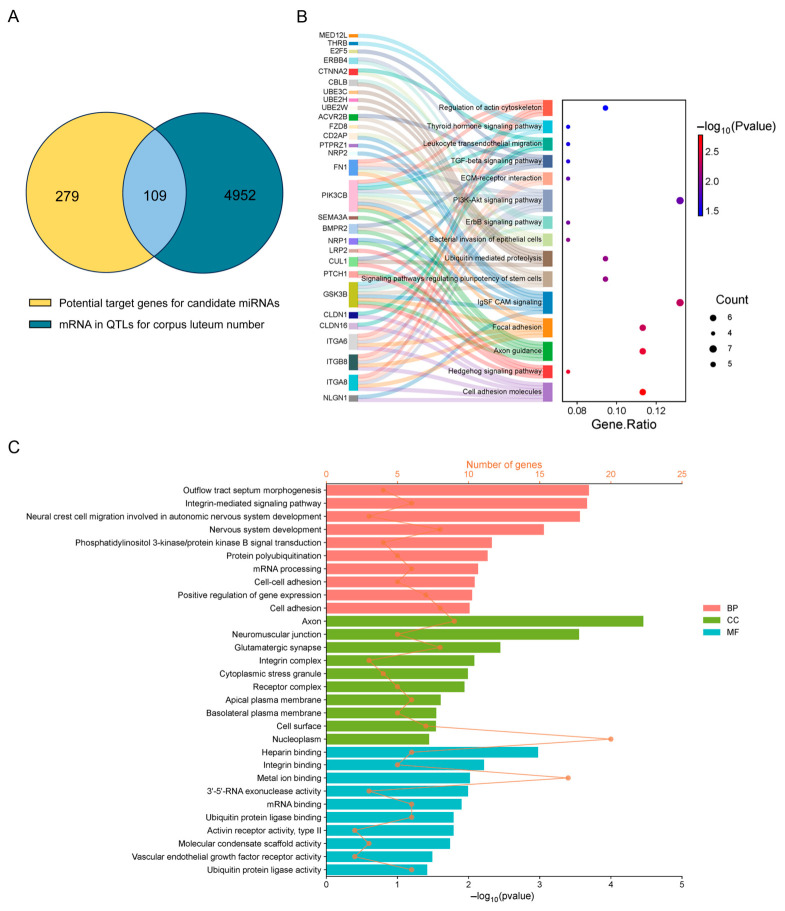
Prediction and function analysis of the miRNA target genes. (**A**) The diagram depicts potential target genes for miRNA binding located in QTLs for corpus luteum number. (**B**,**C**) Potential functions and enriched signaling pathways of the cytoplasmally overexpressed miRNAs were analyzed by KEGG (**B**) and GO (**C**) analyses. The columns in green, blue, and red indicate CC (cell component), MF (molecular function), and BP (biological process) categories.

As negative post-transcriptional regulators, miRNAs typically function by binding to complementary sequences in the 3’UTR of target mRNAs [20]. To investigate the potential regulatory roles of the candidate miRNAs identified in this study, we predicted their target genes using TargetScan and miRDB. This analysis yielded 109 common target genes that were predicted by both databases (the overlapping target genes were screened between 2453 targets predicted by TargetScan and 1401 targets obtained from miRDB) and were also localized within QTL for corpus luteum number (Figure 4A, Table S4). KEGG and GO functional enrichment analyses were performed on the aforementioned target genes. The results revealed that 22 pathways significantly enriched were associated with disease onset and signal transduction (IgSF CAM signaling, ErbB signaling pathway, PI3K-Akt signaling pathway, TGF-beta signaling pathway, thyroid hormone signaling pathway) (Figure 4B). Simultaneously, GO analysis identified 75 significantly enriched functional entries, including processes such as protein modification (protein polyubiquitination), gene expression regulation (mRNA processing, positive regulation of gene expression), and enzyme activity (vascular endothelial growth factor receptor activity, ubiquitin protein ligase activity) (Figure 4C). Notably, several enriched signaling pathways have been previously demonstrated to participate in regulating the reproductive processes of sows.

### 3.5. The TF-lncRNA Interactions

**Figure 5:**
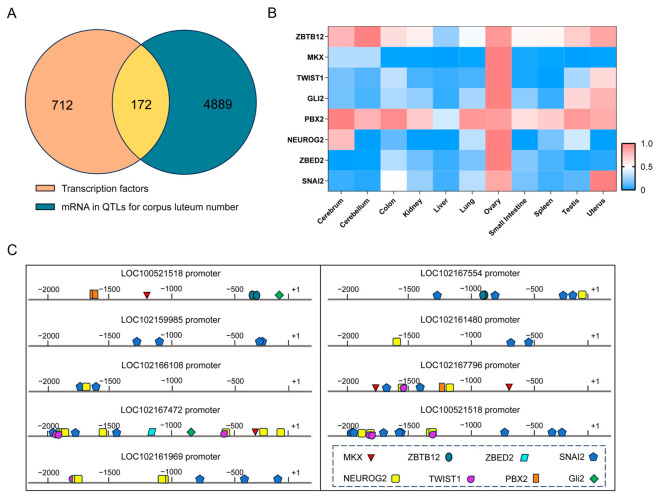
TF-lncRNA interactions. (**A**) Diagram depicting TFs located in QTLs for corpus luteum number. (**B**) Tissue expression profile of ovarian highly expressed TFs. TF tissue expression data are sourced from NCBI Gene Expression Omnibus [21]. (**C**) Schematic diagram of TF-lncRNA binding. Potential binding TFs in lncRNA promoter regions predicted using JASPAR (https://jaspar.elixir.no/).

To investigate TF-lncRNA interaction pairs that may play key regulatory roles during ovulation, TFs located within QTLs for corpus luteum number were first identified, yielding a total of 172 candidates (Figure 5A). Further analysis of tissue expression profiles revealed that eight of these TFs exhibited high expression in ovarian tissue (Figure 5B), suggesting their potential role in regulating ovarian function. To investigate the regulatory relationships between these candidate TFs and lncRNAs, we performed TF binding site prediction analysis using the JASPAR database. The results revealed that the promoter regions of lncRNAs were found to contain binding motifs for multiple distinct TFs, and concurrently, individual TFs were predicted to target several lncRNAs (Figure 5C). This observed many-to-many relationship suggests that the expression of ovulation-related genes is likely governed by the coordinated action of multiple TFs, rather than the independent function of any single factor.

### 3.6. The Construction of the TF-lncRNA-miRNA-mRNA Network

**Figure 6:**
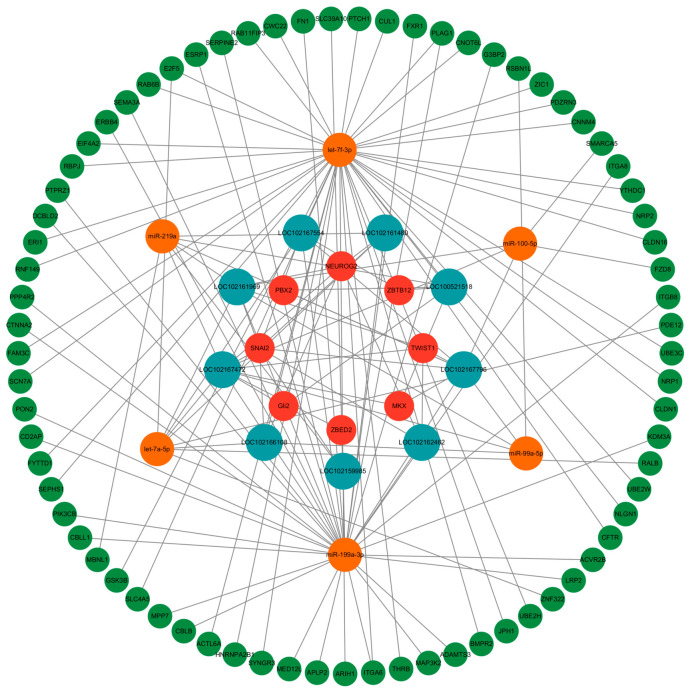
The construction of the TF-lncRNA-miRNA-mRNA network. The circles in red, blue, green, and yellow indicate TFs, lncRNAs, miRNAs, and mRNAs.

To elucidate the regulatory interplay among TFs, lncRNAs, miRNAs, and mRNAs within QTLs for corpus luteum number, an interaction network was constructed (Figure 6). This network, built by integrating the predicted regulatory relationships between these molecular layers, consists of 96 nodes, including eight transcription factors, nine lncRNAs, six miRNAs, and 73 mRNAs.

### 3.7. Gene Correlation Analysis in Sow Follicles

**Figure 7:**
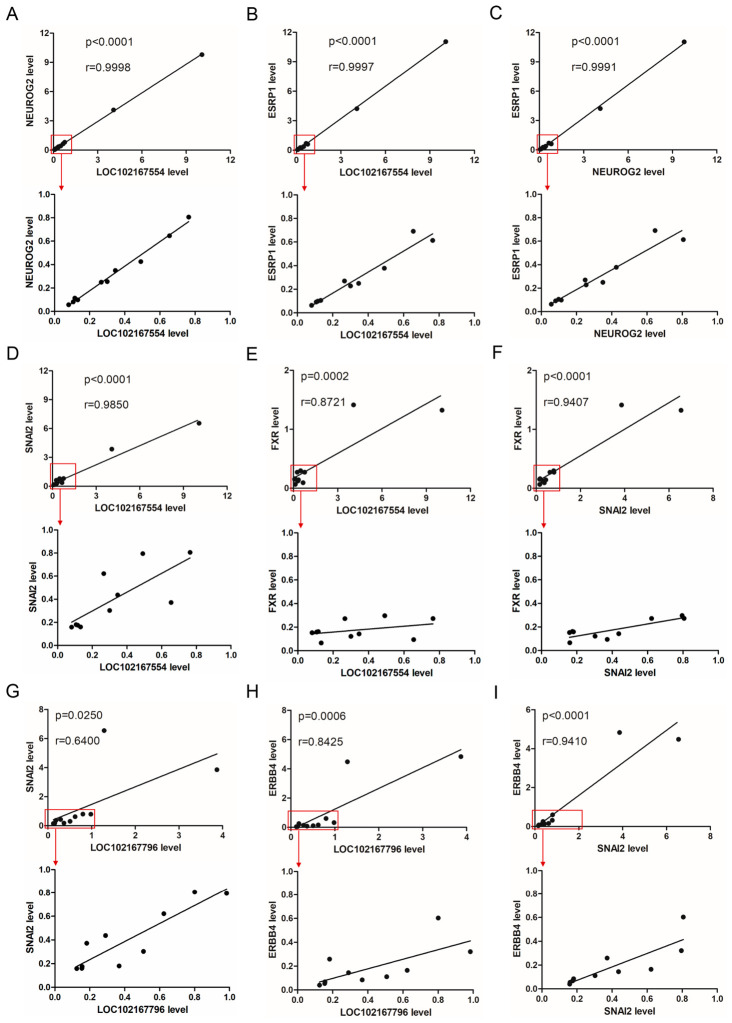
Gene correlation analysis in sow follicles. (**A**) *NEUROG2* vs. *LOC102167554*. (**B**) *ESRP1* vs. *LOC102167554*. (**C**) *ESRP1* vs. *NEUROG2*. (**D**) *SNAI2* vs. *LOC102167554*. (**E**) *FXR* vs. *LOC102167554*. (**F**) *FXR* vs. *SNAI2*. (**G**) *SNAI2* vs. *LOC102167796*. (**H**) *ERBB4* vs. *LOC102167796*. (**I**) *ERBB4* vs. *SNAI2*. *n*  =  12.

To assess the reliability and accuracy of the constructed molecular interaction network, several key node genes were randomly selected for expression correlation validation. The results demonstrated a statistically significant positive correlation in expression levels among the transcription factor *NEUROG2*, lncRNA *LOC102167554*, and its potential target gene *ESRP1* (*p* < 0.0001) (Figure 7A–C). These findings suggest the possibility of synergistic regulatory mechanisms or functional interactions among these molecules in vivo. Furthermore, significant positive correlations were identified between the transcription factor *SNAI2*, the lncRNA *LOC102167554*, and its predicted downstream target gene *FXR* (Figure 7D–F). A similar correlation pattern was also observed involving *SNAI2*, another lncRNA, *LOC102167796*, and its potential target gene *ERBB4* (Figure 7G–I). These findings are in line with established regulatory paradigms. *NEUROG2* and *SNAI2* promote downstream gene expression [22,23,24], and lncRNAs exert positive regulatory effects on targets, often through ceRNA mechanisms [25]. The consistency of our validation with these expected directions offers preliminary experimental support for the biological relevance of the proposed molecular interaction network.

### 3.8. LOC102167554 Serves as a ceRNA for miR-199a-3p and Regulates sGC Proliferation

**Figure 8:**
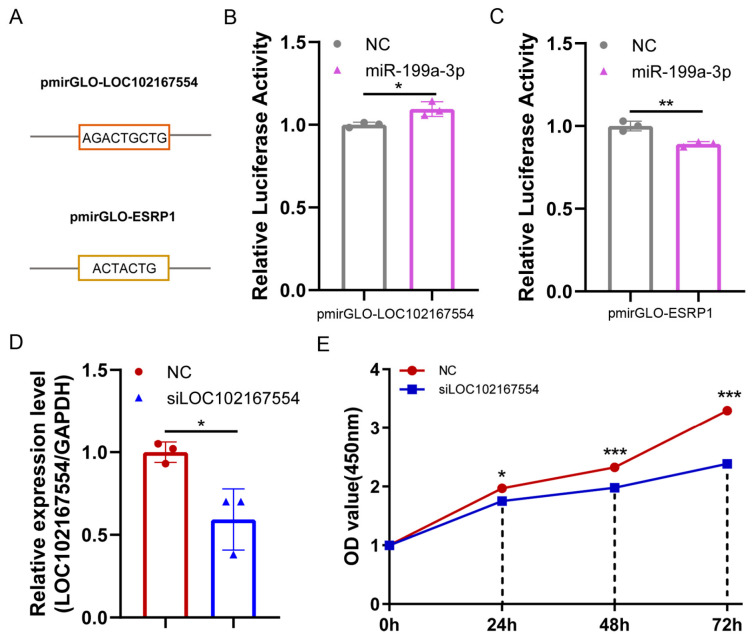
*LOC102167554* serves as a ceRNA for miR-199a-3p and regulates sGC proliferation. (**A**) Schematic representation of the reporter constructs of the *LOC102167554* and *ERBP1* transcripts harboring miR-199a-3p motifs. (**B**,**C**) Luciferase activity was measured in HEK-293T co-transfected with miR-199a-3p mimics and reporter constructs of the *LOC102167554* (**B**) and *ERBP1* (**C**) transcripts harboring the motif. *n* = 3. (**D**) sGCs were transfected with siLOC102167554, and *LOC102167554* levels were detected. *n* = 3. (**E**) After transfection with siLOC102167554, the proliferative capacity of sGCs was assessed. *n* = 6. Quantitative data are plotted as mean ± standard error. * *p* < 0.05. ** *p* < 0.01. *** *p* < 0.001.

To further explore the authenticity of the screened regulatory axis, we selected the *LOC102167554*/miR-199a-3p/*ESRP1* axis for validation. Reporter plasmids of *LOC102167554* and *ESRP1* transcripts containing miR-199a-3p binding sites were constructed (Figure 8A). After transfection of miR-199a-3p mimics into HEK-293T cells, the luciferase activity of the *LOC102167554* transcript reporter plasmid harboring miR-199a-3p binding sites was significantly upregulated (Figure 8B), while the activity of the *ESRP1* transcript reporter plasmid containing miR-199a-3p binding sites was significantly downregulated (Figure 8C). Collectively, these data indicate that miR-199a-3p can bind to both *LOC102167554* and *ESRP1* transcripts, and *LOC102167554* may function as a ceRNA for *ESRP1*.

To further confirm whether *LOC102167554* is a functional lncRNA, siRNA targeting *LOC102167554* (siLOC102167554) was synthesized. After transfection of siLOC102167554 into sGCs, the expression level of *LOC102167554* was significantly downregulated (Figure 8D). Notably, knockdown of *LOC102167554* significantly inhibited the proliferation of sGCs. In conclusion, *LOC102167554* serves as a ceRNA for miR-199a-3p and regulates sGC proliferation.

## 4. Discussion

Ovulation number is a critical determinant of reproductive performance in sows. As a typical quantitative trait, it is governed by a complex polygenic regulatory network. To date, several key genes influencing ovulation have been identified, including *STAT3*, *ISG15*, and *GPR43* [26,27,28]. Furthermore, advances in genome-wide association studies and high-throughput sequencing technologies have led to the successful localization of multiple QTLs significantly associated with ovulation number (corpus luteum number) in sows [1,29,30]. These QTLs define genomic intervals containing a wealth of potential candidate genes. Notably, sow ovulation, as a complex quantitative trait, is controlled by numerous minor-effect loci with non-linear interaction and modular regulatory characteristics, which cannot be fully interpreted by traditional linear single-gene models. Nevertheless, the systematic elucidation of functional genes within these regions and the analysis of their regulatory networks remain underexplored. The present study was designed to rigorously screen candidate genes within QTLs associated with ovulation traits, aiming to pinpoint key regulators of sow ovulation. To this end, we constructed a molecular regulatory network comprising TF-lncRNA-miRNA-mRNA.

As important epigenetic regulators, lncRNAs are extensively involved in various cellular biological processes, including those closely associated with reproductive traits in sows, such as the regulation of ovarian granulosa cell apoptosis, E2 synthesis, and follicular development [31,32]. This study, starting from lncRNAs, identified that 36.3% of reproduction-related lncRNAs are located within QTLs for corpus luteum number. Among these, nine lncRNAs are highly expressed in follicles and are primarily localized in the cytoplasm. In the cytoplasm, lncRNAs primarily function as ceRNAs by binding to miRNAs, thereby inhibiting the binding of miRNAs to their target genes. This enhances the stability of target mRNA and promotes its expression [33]. Consequently, the nine lncRNAs may exert their functions through the ceRNA mechanism.

miRNAs are widely recognized as participating in various cellular biological processes, including cell proliferation, differentiation, apoptosis, and disease pathogenesis, which are often regulated by lncRNAs. In this study, using RNAhybrid prediction, we identified six potential miRNAs capable of binding to the nine candidate lncRNAs. Notably, the regulatory interactions between these lncRNAs and miRNAs are not restricted to a simple one-to-one correspondence but rather form a complex and intricate network. Specifically, several key lncRNAs identified in this study, including *LOC102167554*, *LOC102167472*, *LOC102161480*, *LOC102161969*, and *LOC102167796*, harbor multiple miRNA binding sites, enabling them to act as ceRNAs that sequester multiple miRNAs simultaneously. Each of the six candidate miRNAs interacts with multiple lncRNAs, indicating a reciprocal and multi-dimensional regulatory pattern between lncRNAs and miRNAs in the context of sow ovulation regulation. Further analysis of these miRNAs revealed that they were not only located within QTLs for corpus luteum number but also exhibited high expression levels in ovarian tissue. Interestingly, multiple candidate miRNAs have been reported to be differentially expressed in reproduction-related processes, with certain members experimentally validated as regulators of ovarian function [34,35,36,37]. GO and KEGG pathway enrichment analyses were performed on the downstream target genes of candidate miRNAs. Functional annotation revealed that these genes may participate in multiple regulatory functions, such as protein modification, gene expression regulation, and enzyme activity. They influence key signaling pathways—including the IgSF CAM, ErbB, PI3K-Akt, and TGF-beta. Notably, the ErbB signaling pathway modulates follicle atresia and oocyte maturation by activating downstream cascades, including PI3K-Akt, thereby directly affecting ovulation capacity and corpus luteum formation in sows [38,39]. As a core reproductive regulatory cascade, the PI3K-Akt signaling pathway contributes to ovarian cell survival, follicular apoptosis inhibition, and hormonal homeostasis, which further stabilizes folliculogenesis and reproductive balance [40,41]. In addition, the TGF-beta signaling superfamily acts as a vital regulatory axis in the ovary and dominates granulosa cell apoptosis, steroid hormone synthesis, and follicle selection [42,43,44]. These multiple signaling pathways synergistically determine the ovulation number of sows.

TFs are pivotal regulators of gene expression that modulate transcription by binding to specific DNA sequences [45]. In this study, potential interactions between TFs and lncRNAs were predicted, leading to the identification of eight TFs located within QTLs for corpus luteum number and exhibiting high expression in ovarian tissue. These TFs, along with the previously screened miRNAs and key pathway-related mRNAs, were integrated to construct a multi-level regulatory network comprising TF–lncRNA–miRNA–mRNA interactions. Hub molecules were subsequently identified, including key transcription factors (*SNAI2* and *NEUROG2*), core lncRNAs (*LOC102167554* and *LOC102167796*), and downstream target genes (*FXR*, *ERBB4*, and *ESRP1*). Notably, *FXR* is mainly localized in secondary and tertiary follicles, especially in granulosa cells, and regulates the expression of genes involved in estrogen synthesis in ovarian granulosa cells [46]. *ERBB4* is expressed in the early corpus luteum and mediates cell proliferation, survival, and chemotaxis [47,48]. *ESRP1* is differentially expressed in ovarian tissues between normal and low-fecundity sows [49]. Collectively, these results indicate that these three genes may act as potential key factors modulating sow ovulation. Furthermore, correlation analysis revealed significant positive correlations between transcription factor *NEUROG2*, lncRNA *LOC102167554*, and its potential downstream target gene *ESRP1*. Similarly, significant positive correlations were also observed between *SNAI2*, *LOC102167554*, and its target gene *FXR*, as well as among *SNAI2*, lncRNA *LOC102167796*, and ERBB4. Previous studies have confirmed that transcription factors *NEUROG2* and *SNAI2* promote the expression of downstream genes [22,23,24] and that lncRNAs positively regulate target gene expression when functioning via the ceRNA mechanism [25]. Using the JASPAR database, we predicted the binding sites of transcription factors *NEUROG2* and *SNAI2* in the promoter of *LOC102167554* and *LOC102167796*. Collectively, we hypothesized that *NEUROG2* and *SNAI2* may act as transcriptional activators to initiate the transcription of the key lncRNAs (*LOC102167554* and *LOC102167796*), thereby regulating reproductive performance in sows. Therefore, based on existing research evidence, we have identified three potential regulatory axes influencing sow ovulation numbers: *NEUROG2*/*LOC102167554*/*ESRP1*, *SNAI2*/*LOC102167554*/*FXR*, and *SNAI2*/*LOC102167796*/*ERBB4*. However, the specific molecular mechanisms remain unclear and require further investigation.

To further verify the authenticity of the screened regulatory axes and clarify the role of miRNAs in the regulatory network, we selected the *LOC102167554*/miR-199a-3p/*ESRP1* axis for functional validation. The results showed that the addition of miR-199a-3p mimic significantly increased the luciferase activity of the reporter plasmid containing the miR-199a-3p binding site of *LOC102167554* while significantly decreasing the luciferase activity of the reporter plasmid containing the miR-199a-3p binding site of *ESRP1*. This is consistent with the result that *LOC102167554*, as a ceRNA, positively regulates *ESRP1* expression, and the two exhibit a significant positive correlation. However, it is generally accepted that miRNAs negatively regulate the expression of their target genes, including lncRNAs. The positive regulation of *LOC102167554* by miR-199a-3p may be attributed to the binding between them, which enhances the stability of *LOC102167554* transcripts rather than inducing their degradation. Previous studies have confirmed that several miRNAs localized in the cytoplasm play an important role in maintaining the stability of target genes [4,50]. In addition, the functional validation results showed that the interference of *LOC102167554* significantly reduced the proliferation ability of sGCs. *ESRP1* has been confirmed to promote the proliferation of various cell types [51,52], and the downregulation of *LOC102167554* expression inhibited *ESRP1* expression, thereby impairing the proliferation of sGCs. This result is consistent with the regulatory mechanism of ceRNA and also aligns with the existing reports on the regulation of cell proliferation by ceRNA-mediated pathways.

## 5. Conclusions

This study successfully constructed a multi-level interactive regulatory network involving TFs, lncRNAs, miRNAs, and mRNAs that correlates with ovulation count. Three potential regulatory axes were identified within this network: *NEUROG2*/*LOC102167554*/*ESRP1*, *SNAI2*/*LOC102167554*/*FXR*, and *SNAI2*/*LOC102167796*/*ERBB4*. The construction of this network not only offers a novel theoretical framework for elucidating the molecular regulatory mechanisms underlying ovulation in sows but also highlights key regulatory nodes with potential applications in the genetic improvement and molecular breeding of reproductive traits. In addition, a functional lncRNA, *LOC102167554*, that regulates the proliferation of sGCs was also identified. It should be noted, however, that the current findings are primarily derived from bioinformatics analyses, and the specific molecular mechanisms of the identified regulatory pathways warrant further experimental validation.

## Data Availability

The original contributions presented in this study are included in the article/Supplementary Material. Further inquiries can be directed to the corresponding author.

## Supplementary Materials

The following supporting information can be downloaded at: https://www.mdpi.com/article/10.3390/ani16111693/s1, Table S1: Primers for qRT-PCR; Table S2: Prediction of subcellular localization of lncRNAs; Table S3: miRNAs located in QTLs for corpus luteum number; Table S4: mRNAs targeted by miRNAs located within QTL for corpus luteum number.

## Abbreviations

The following abbreviations are used in this manuscript:

QTL	Quantitative trait loci
TFs	Transcription factors
LncRNAs	Long non-coding RNAs
miRNAs	microRNAs
qRT-PCR	Quantitative real-time PCR
E2	Estradiol
miRNAs	3′ untranslated region
ceRNA	Competing endogenous RNA
KEGG	Kyoto Encyclopedia of Genes and Genomes
GO	Gene ontology
MREs	miRNA response elements
CC	Cell component
MF	Molecular function
BP	Biological process
sGCs	Sow granulosa cells
